# Supplementary figures and images for: Interlocked transcription factor feedback loops maintain and restore touch sensation
Source: bioRxiv. 2025 May 20:2025.05.15.654349. Preprint. [Version 1] doi: 10.1101/2025.05.15.654349 (PMC12139737; doi:10.1101/2025.05.15.654349)

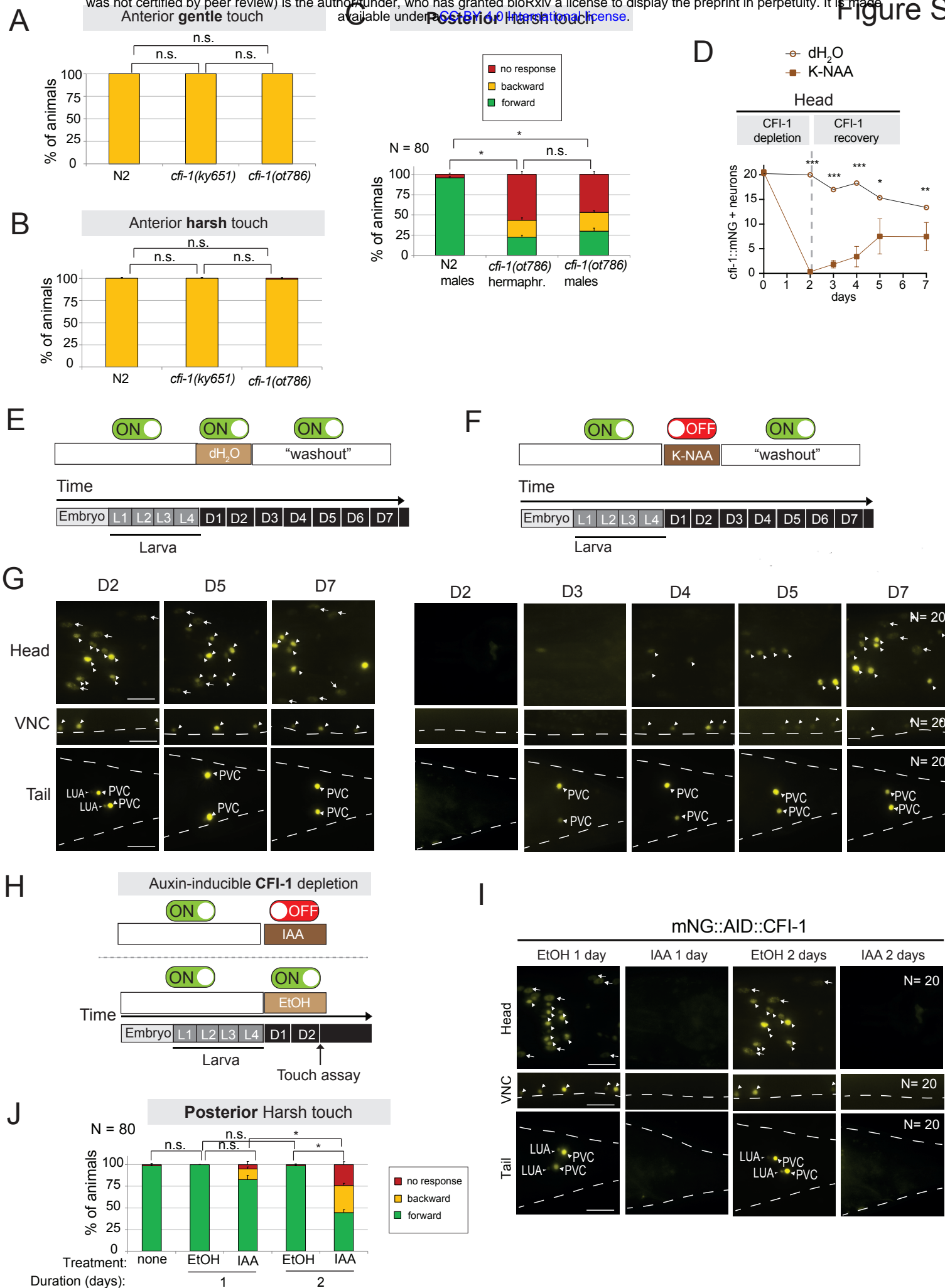

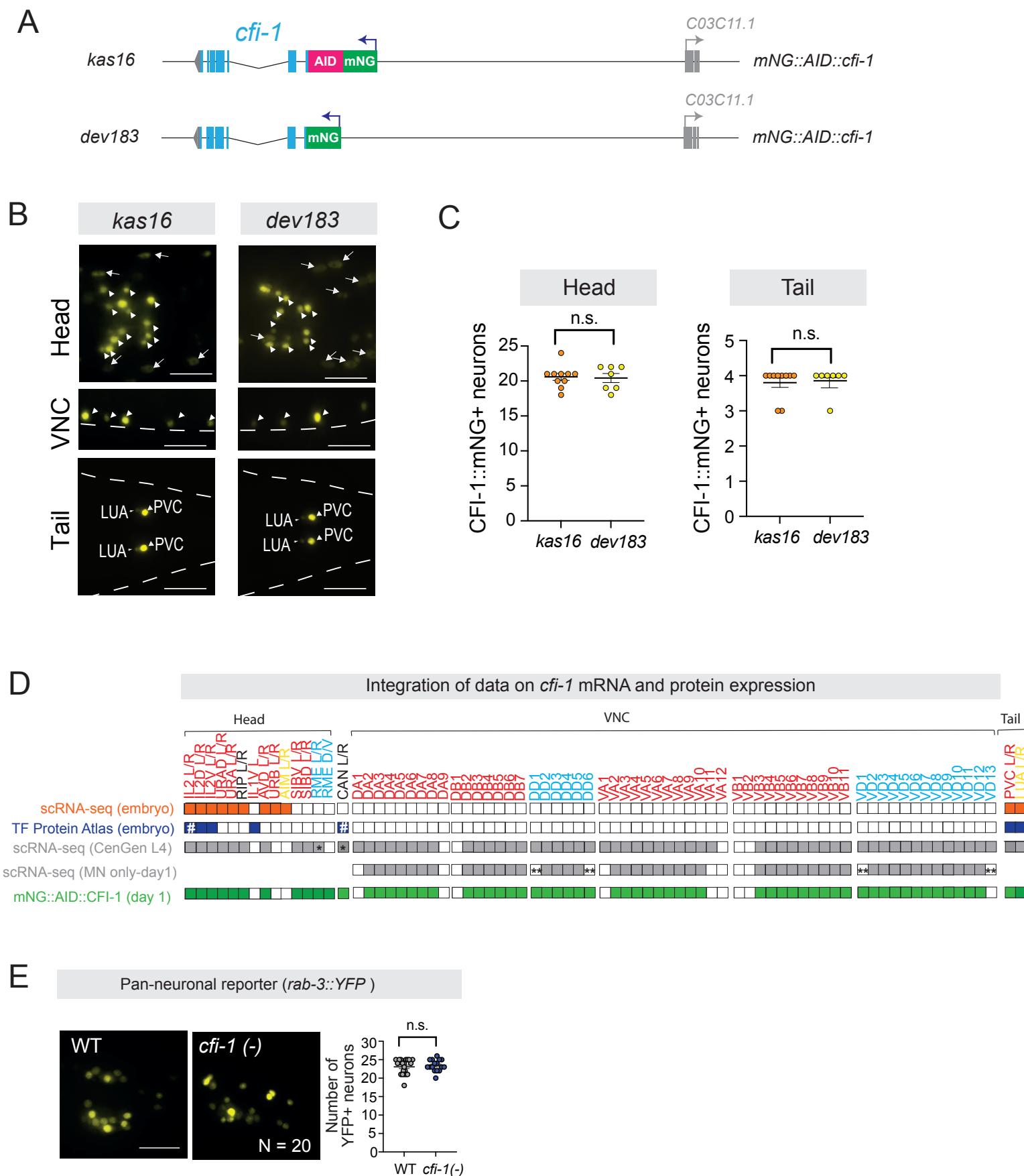

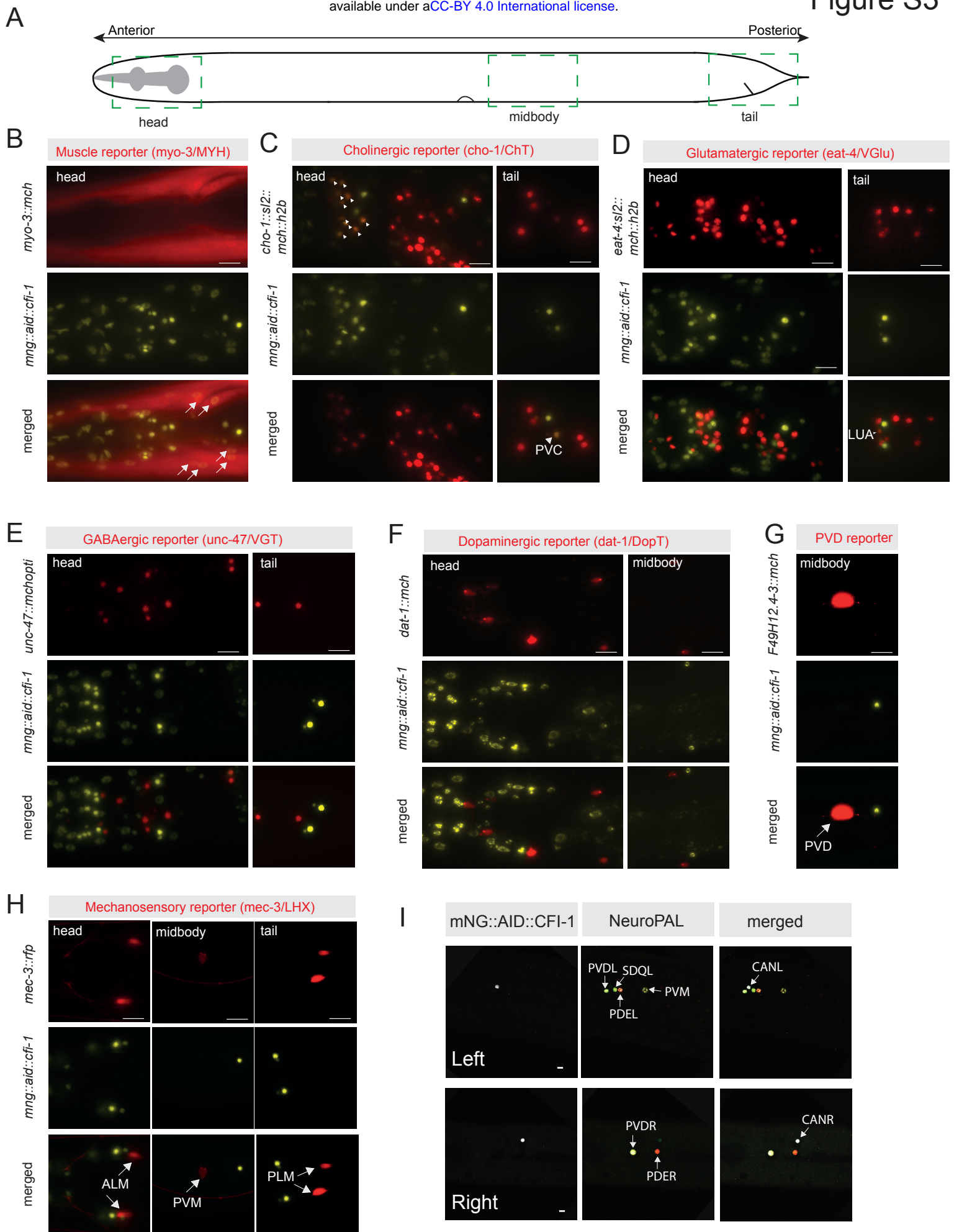

A

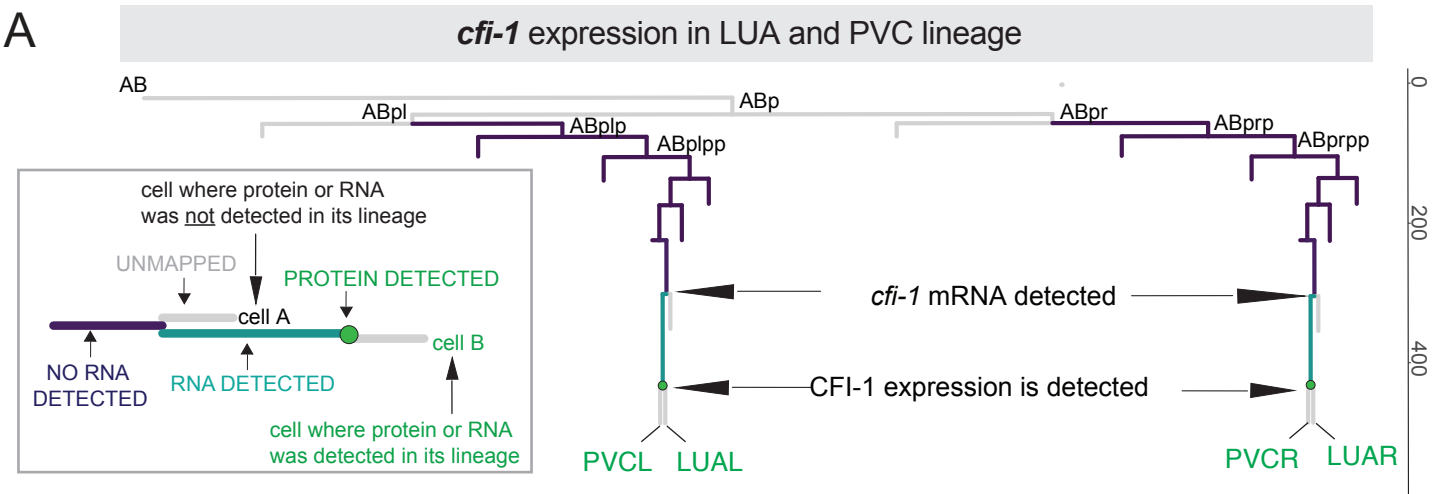

B

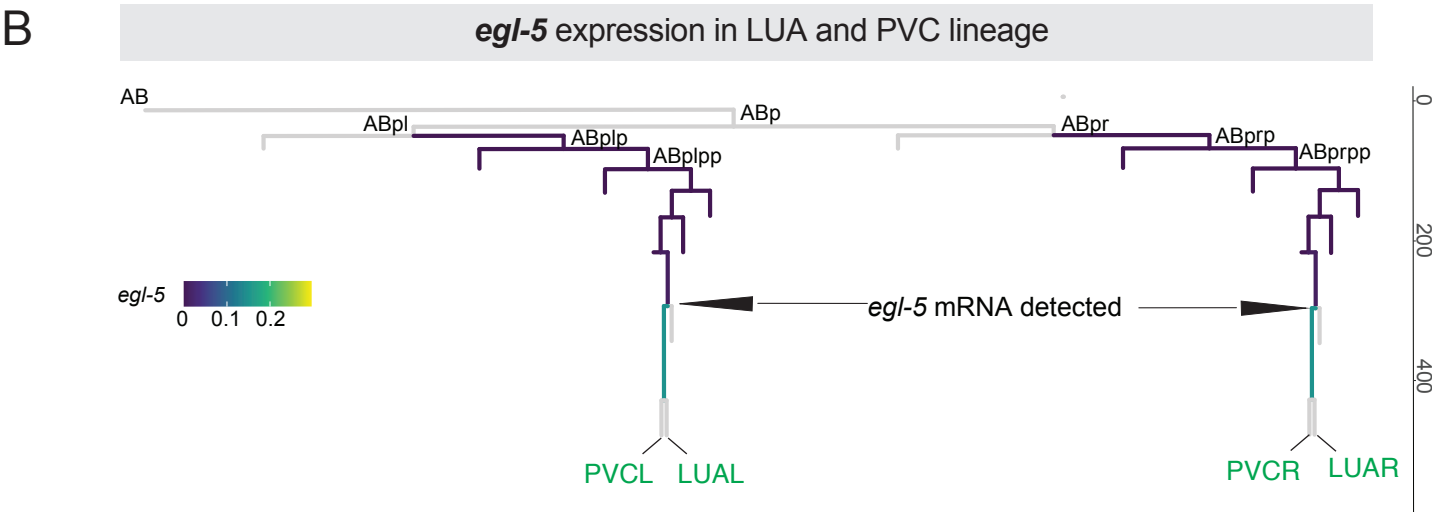

C

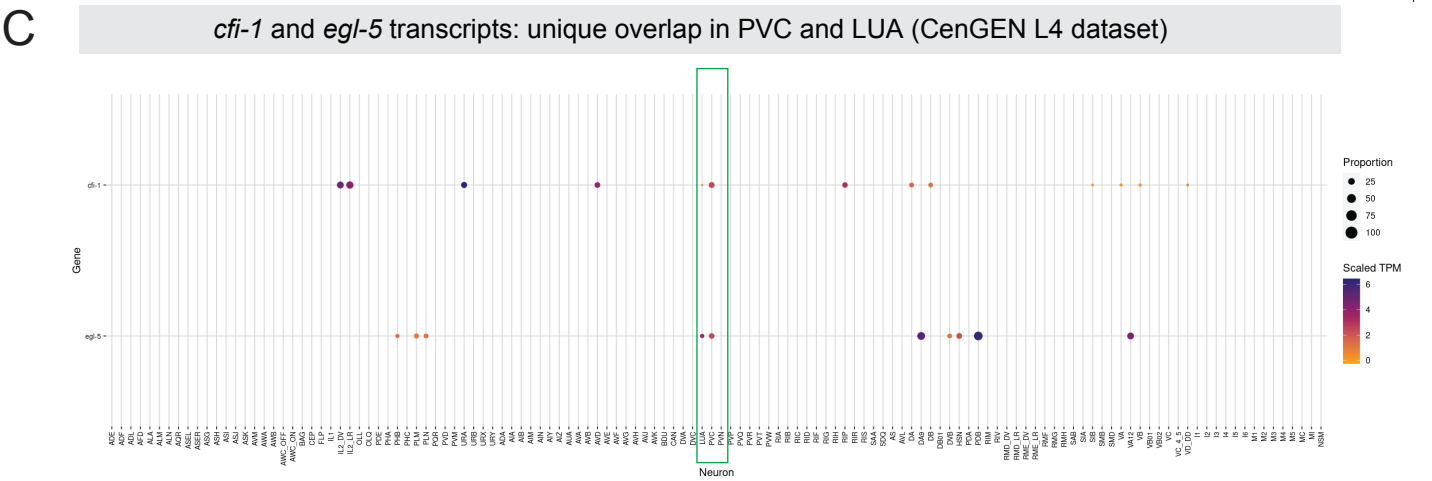

D

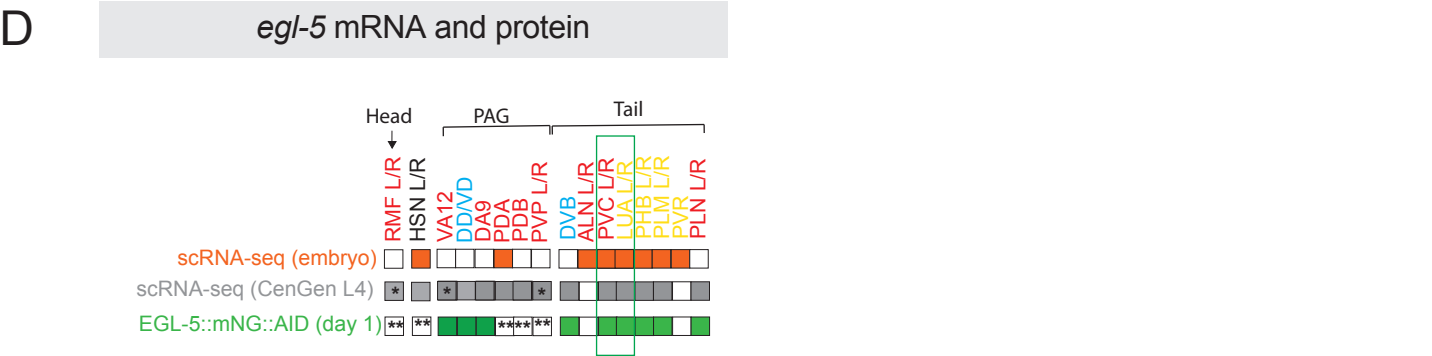

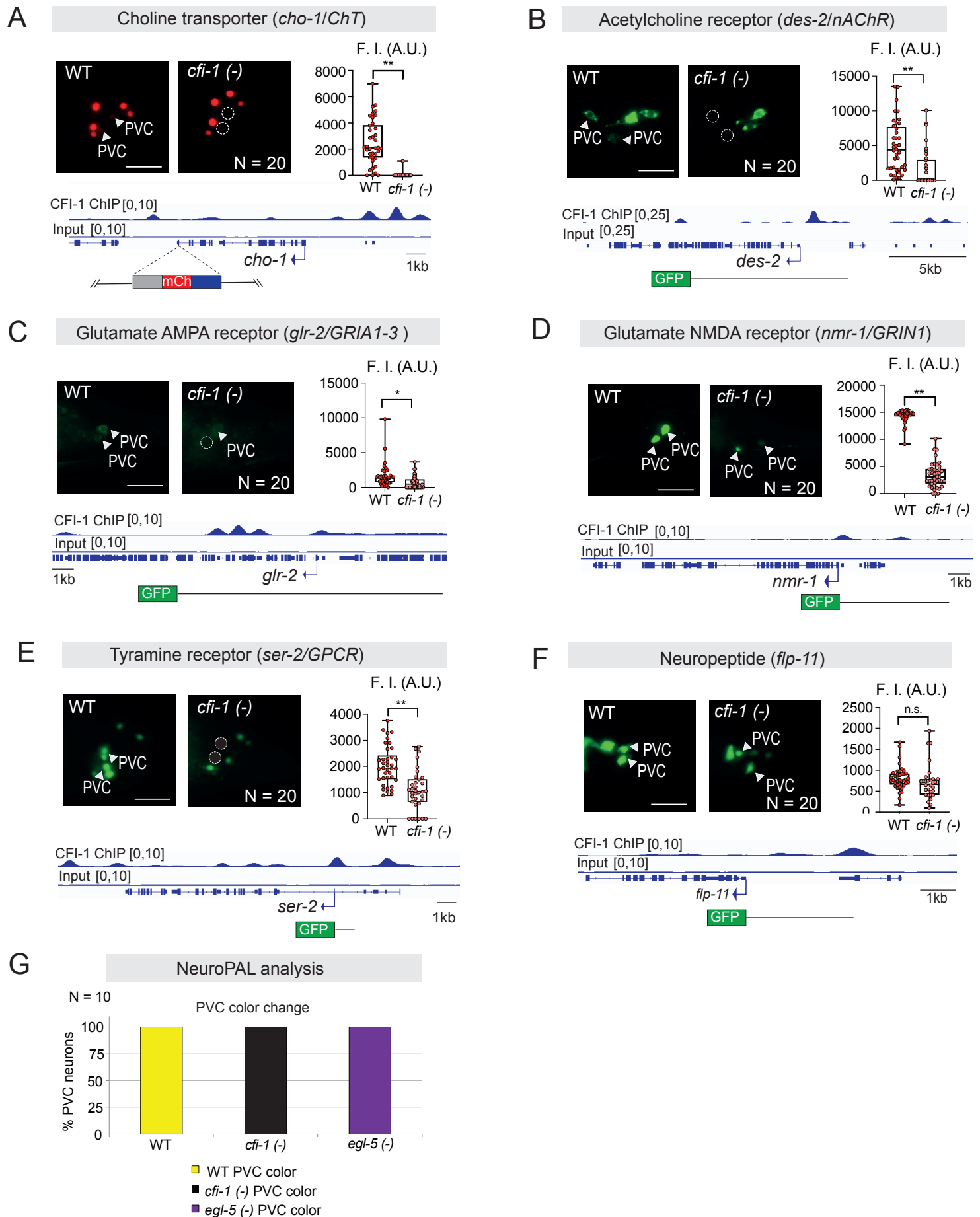

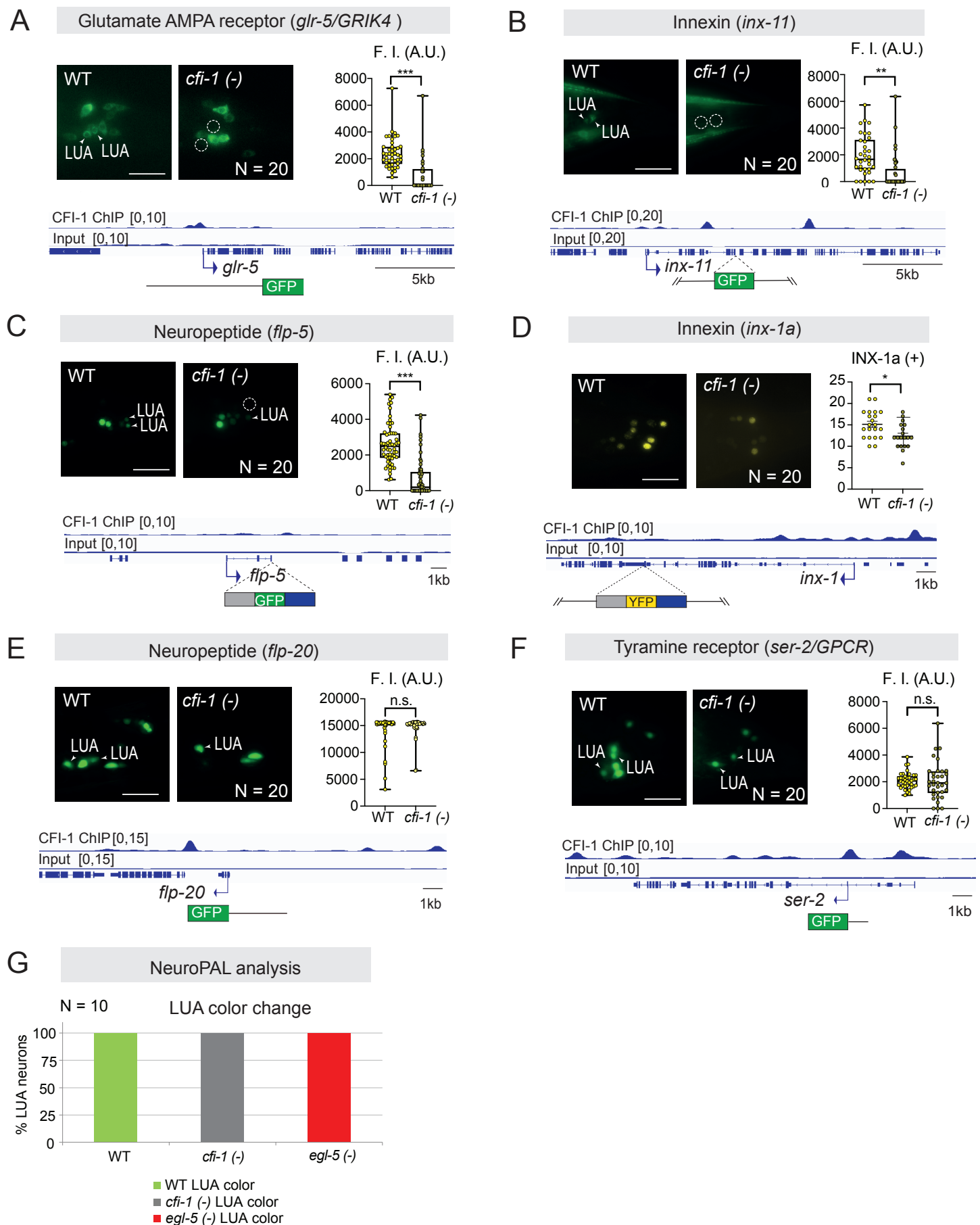

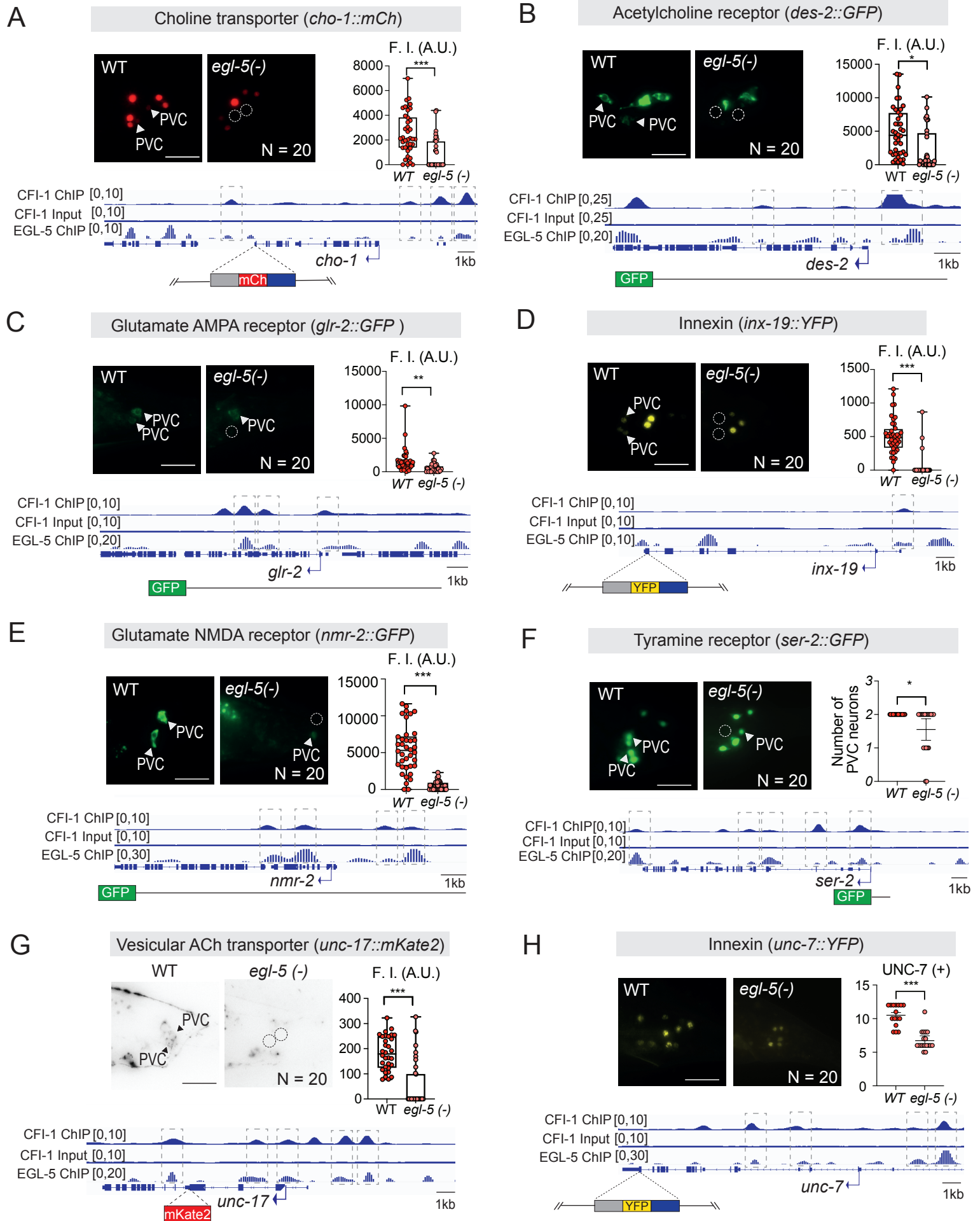

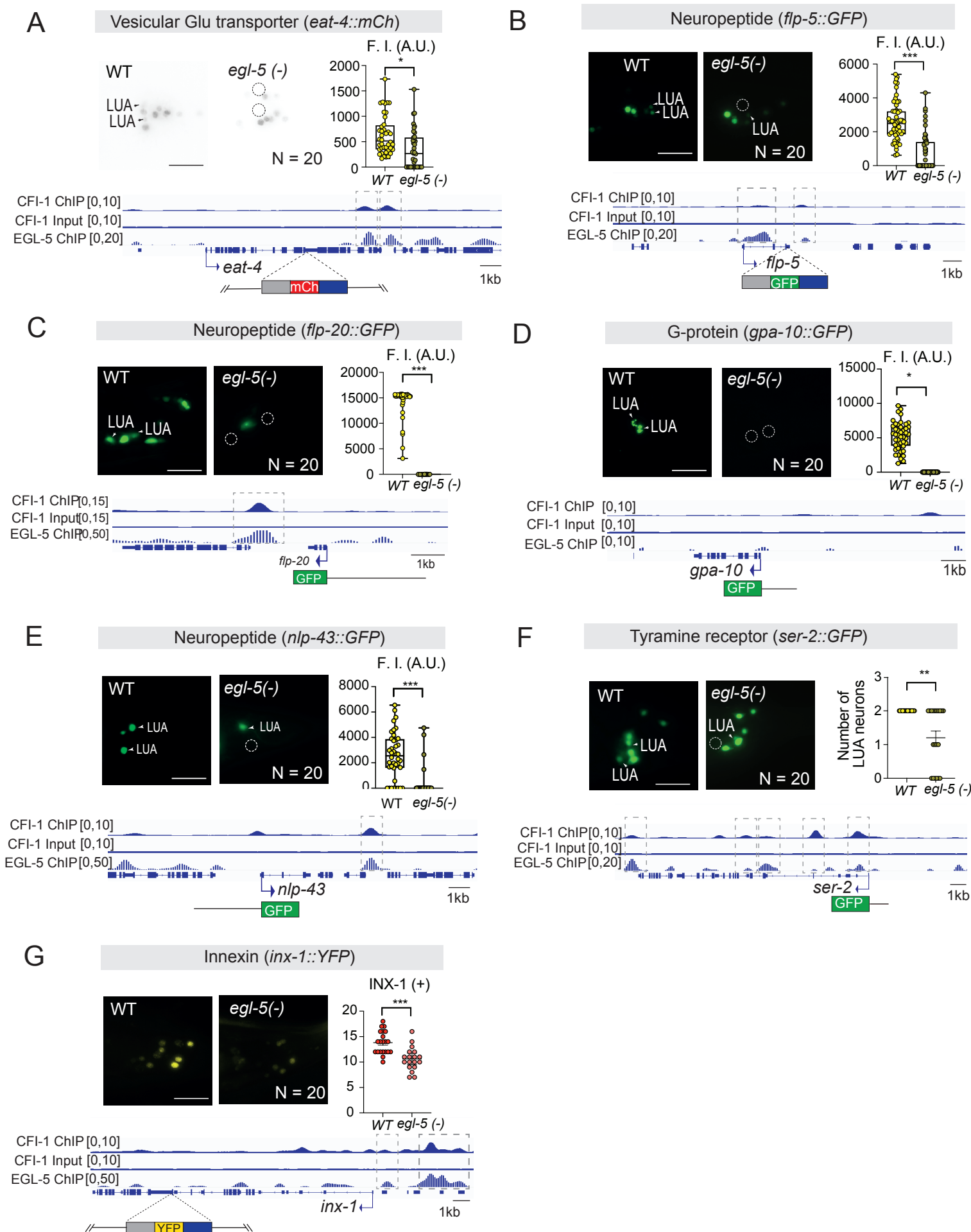

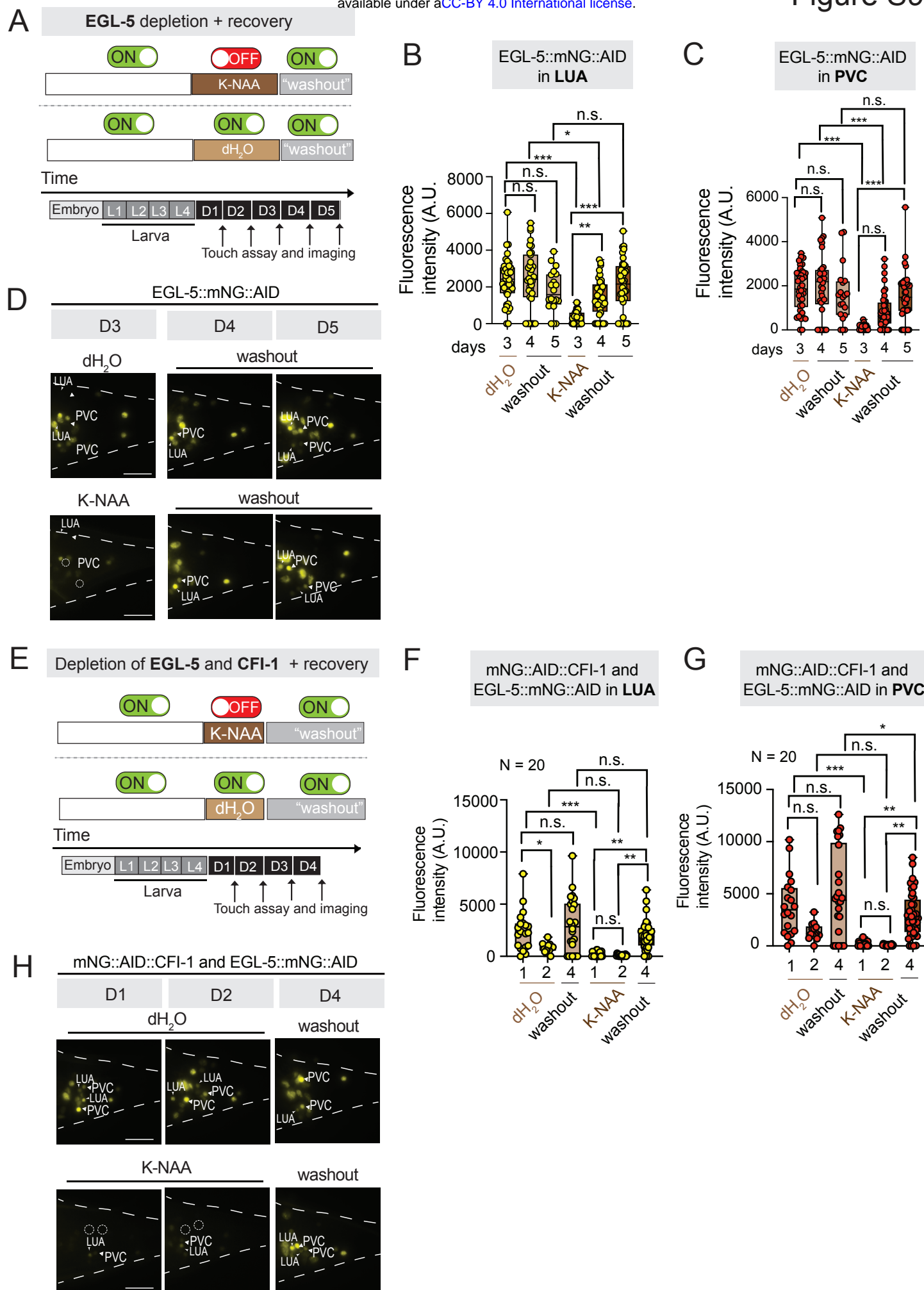

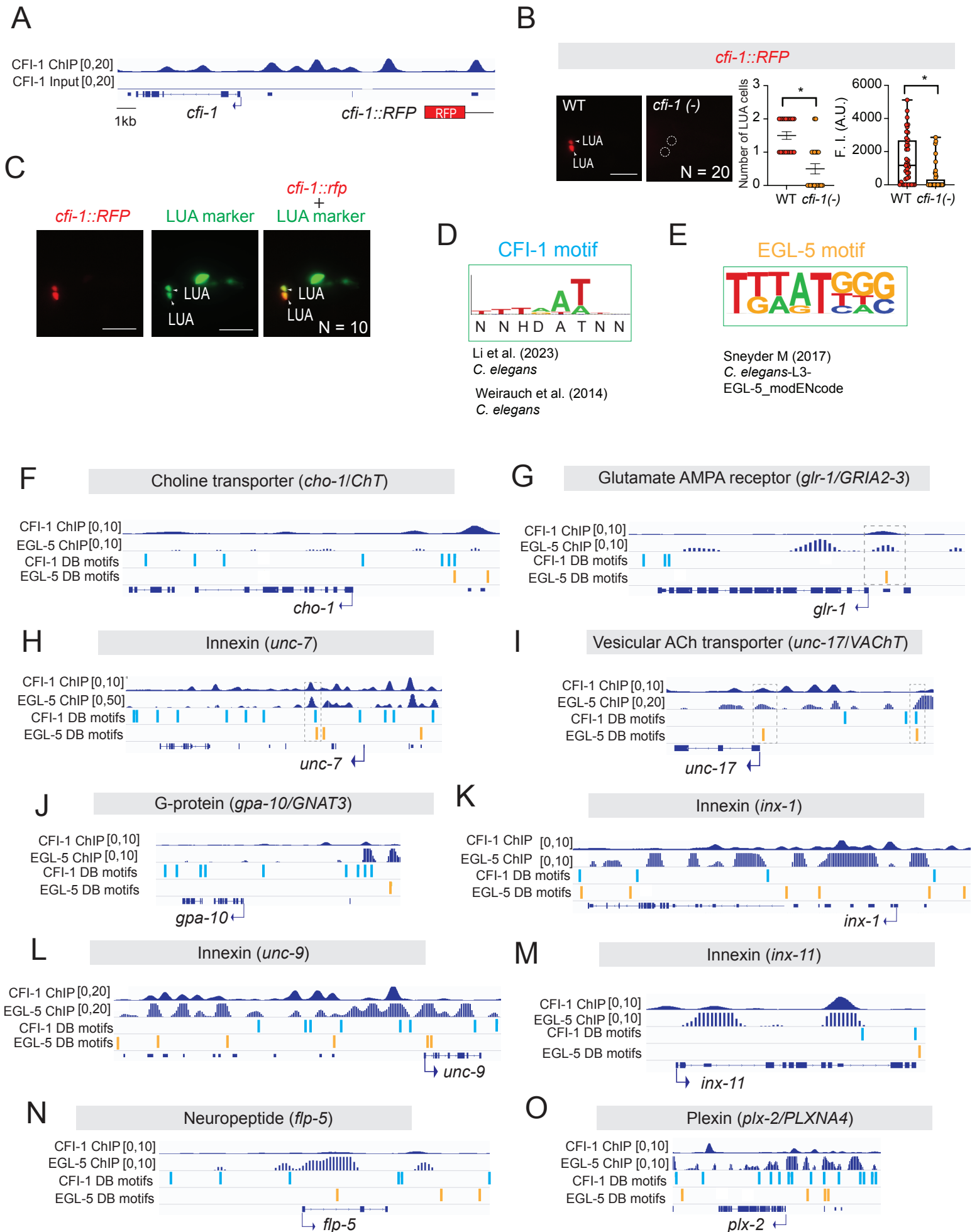

Supplement: 1 — Figure S1: CFI-1 is required to maintain posterior harsh touch responses. A. Anterior gentle touch assay in C. elegans animals carrying cfi-1 mutations. N = 80 animals. B. Anterior harsh touch assay in C. elegans animals carrying cfi-1 mutations. N = 80 animals. C. Posterior harsh touch assay in cfi-1(ot786) mutant animals. Comparison between hermaphrodites and males. N = 80 animals. D. Quantification of head neurons expressing the endogenous CFI-1 reporter after water (control), auxin and “washout” recovery treatments on kas16[mNG::AID::cfi-1]; ieSi57 [eft-3p::TIR1::mRuby::unc-54 3’UTR + Cbr-unc-119(+)] animals. N = 5 animals. E. Diagrams illustrating the timeline of the auxin (K-NAA) and “washout” treatments used for the depletion of CFI-1. F. Diagrams illustrating the timeline of the auxin (K-NAA) and “washout” treatments. G. Recovery of mNG::AID::CFI-1 expression upon auxin treatment followed by washout. Representative images showing mNG::AID::CFI-1 expression in the head, ventral nerve cord (VNC) and tail of C. elegans animals. Arrowheads point to neurons, while the arrows point to head muscle cells. N = 20 animals. H. Diagram illustrating the timeline of the EtOH (control) and auxin (IAA) treatments used for the depletion of CFI-1. I. Representative images showing the loss of mNG::AID::CFI-1 expression in the head, VNC and tail of C. elegans animals. Arrowheads point to neurons, while the arrows point to head muscle cells. N = 20 animals. J. Posterior harsh touch assay in C. elegans animals before and after EtOH (control) and auxin (IAA) treatments for 1 and 2 days. N = 80 animals. For behavior in panels A, B, C and J: *, p < 0.0001; n.s., not significant, as determined by Bonferroni post-hoc tests. For quantifications in panel D: *, p = 0.002; **, p = 0.00003; ***, p < 0.000003 versus auxin treatment, as determined by Bonferroni post-hoc test. Figure S2: CFI-1 expression analysis using endogenous reporters and available RNA-Seq datasets. A. Diagram showing th [file NIHPP2025.05.15.654349v1-supplement-1.pdf]
